# Supplementary figures and images for: TRIM32 inhibits Venezuelan equine encephalitis virus infection by targeting a late step in viral entry
Source: PLoS Pathog. 2024 Nov 11;20(11):e1012312. doi: 10.1371/journal.ppat.1012312 (PMC11581401; doi:10.1371/journal.ppat.1012312)

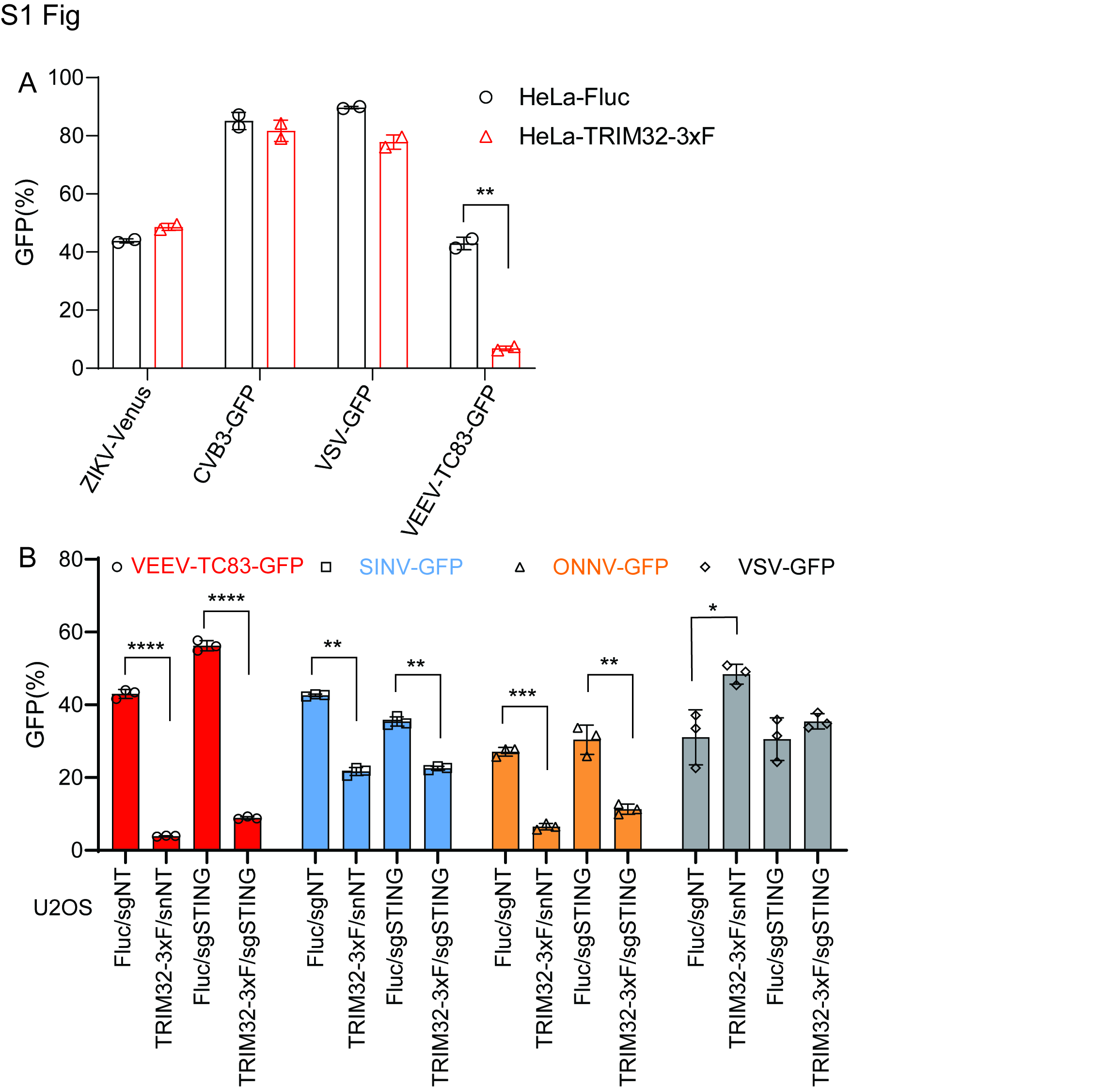

Supplement: S1 Fig — A. HeLa-Fluc or HeLa-TRIM32-3F were infected with the indicated viruses, and viral infectivity was quantified by flow cytometry. B. STING was silenced in U2OS-TRIM32 or U2OS-Fluc cells. Cells were infected with the indicated viruses, and virus infectivity was quantified by flow cytometry. Statistical significance was determined by unpaired students’ t-test for A and B (*P<0.05, **P<0.01, ***P<0.001, ****P<0.0001). (TIF) [file ppat.1012312.s001.tif]

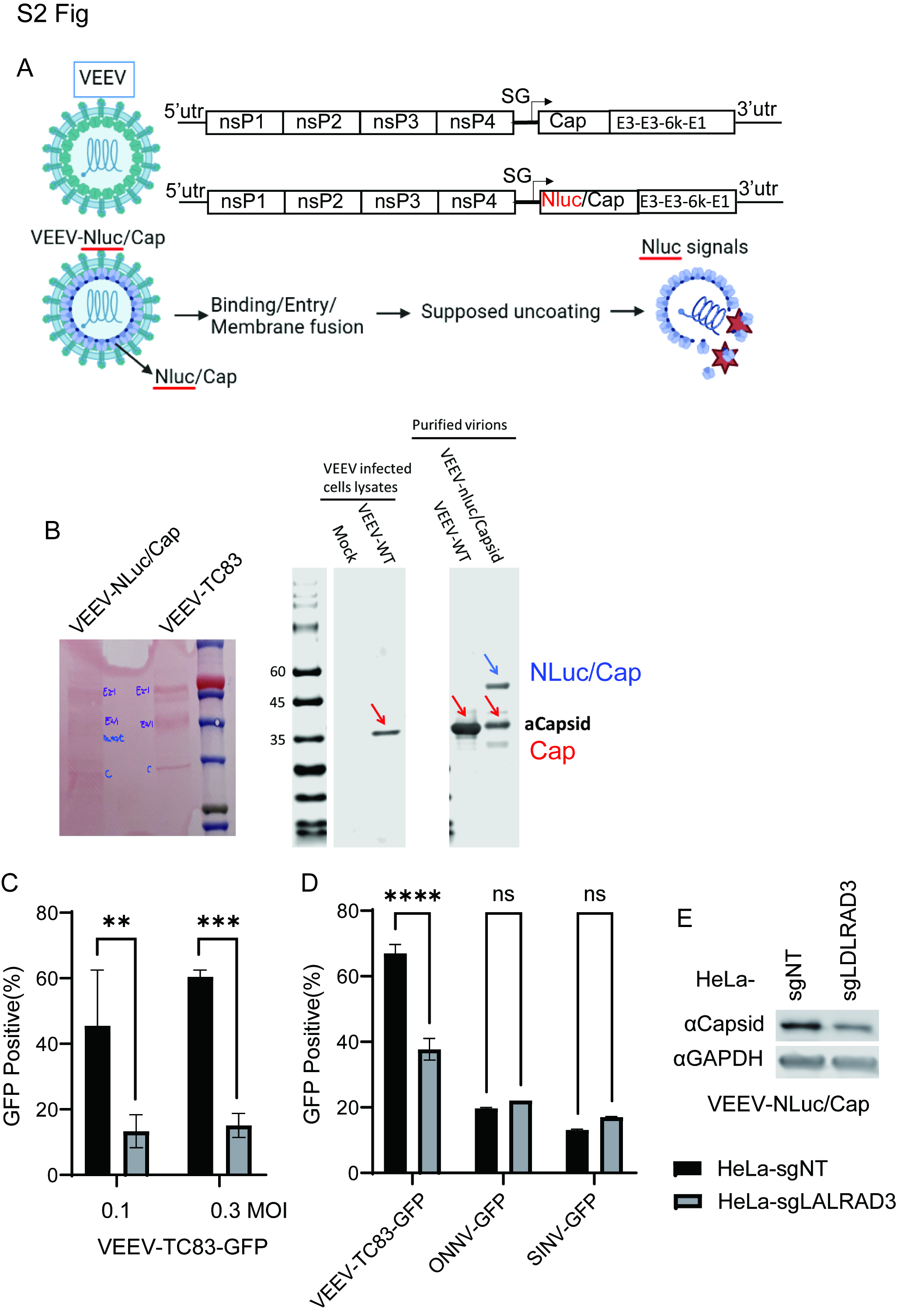

Supplement: S2 Fig — A. Illustration of VEEV-NLuc/Cap. B. VEEV-TC83 and VEEV-NLuc/Cap was purified by ultracentrifugation, and virion proteins were visualized by ponceau S staining and Western blot analysis using antibody against viral capsid protein. C. HeLa cells stable expressing dual-sgRNA targeting VEEV receptor LDLRAD3 or non-specific targeting sgRNA were infected VEEV-TC83-GFP at the indicated MOI for 24h, and virus infectivity was quantified by flow cytometry. D. HeLa cells stable expressing dual-sgRNA targeting VEEV receptor LDLRAD3 or non-specific targeting sgRNA were infected VEEV-TC83-GFP, SINV-GFP, and ONNV-GFP at MOI of 0.1 for 24h, and virus infectivity was quantified by flow cytometry. Statistical significance was determined by unpaired students’ t-test for C and D (**P<0.01, ***P<0.001, ****P<0.0001). ns, no significant. (TIF) [file ppat.1012312.s002.tif]

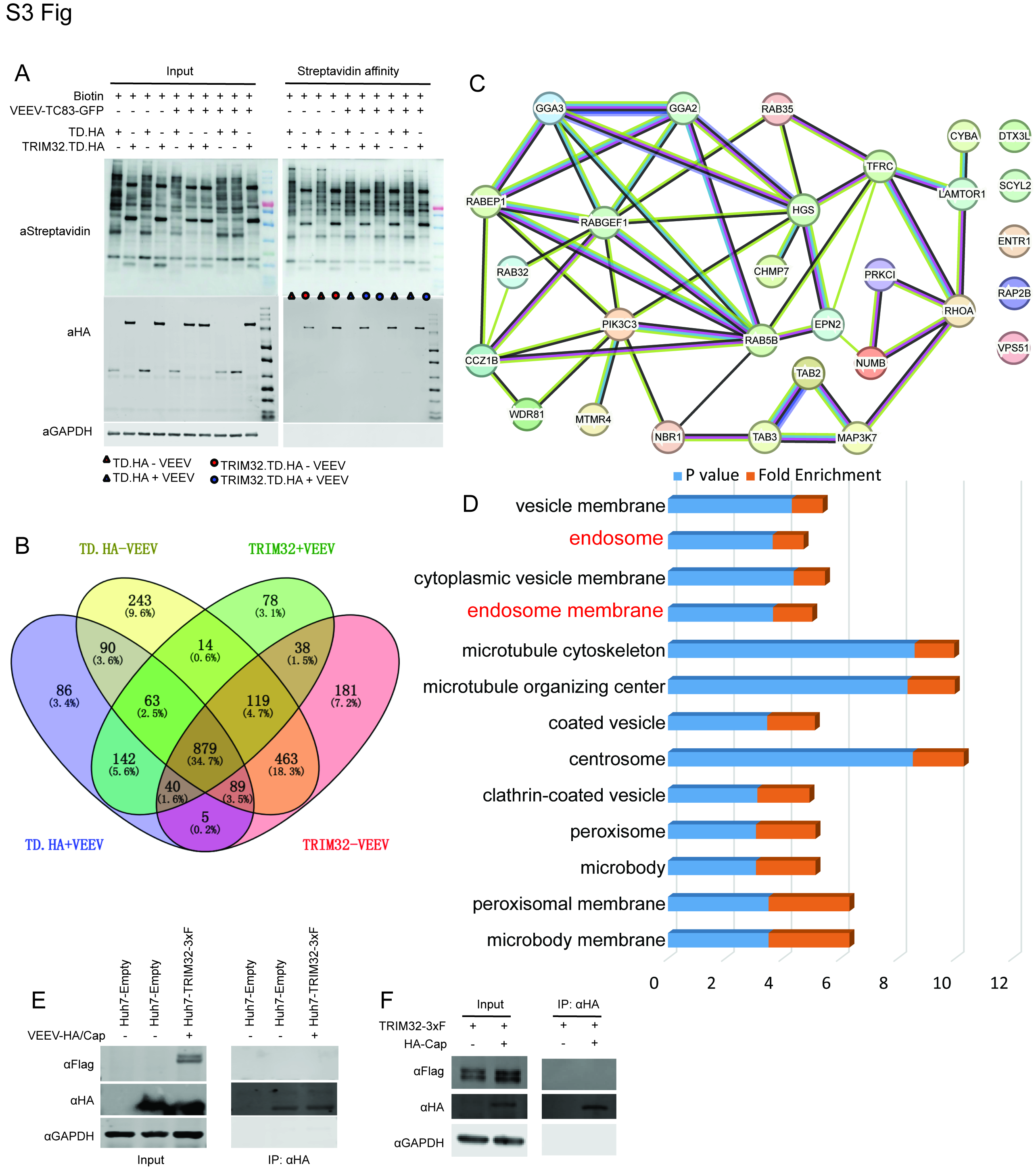

Supplement: S3 Fig — A. Western blot analysis of TRIM32-TurboID system. HeLa-TRIM32-TurboID-HA or HeLa-TurboID-HA cells were infected with or without VEEV-TC83-GFP at MOI of 25 for 6 hrs in the presence of biotin at a concentration of 500μM. The biotinylated proteins were purified by using Streptavidin MagBeads. B. The enriched proteins in each group were analyzed by using Venny 2.1 (Venny 2.1.0 (csic.es). C. The cellular component analysis of TRIM32-enriched proteins by using PANTHER. D. The network of TRIM32 proximity labeled endosome associated proteins. E and F. The interaction between TRIM32 and VEEV capsid was determined by co-immunoprecipitation by using anti-HA beads. In E, Huh7-Empty and Huh7-TRIM32-3xF cells were infected with or without VEEV-HA/Cap at MOI of 50. The cells were collected at 3 hpi, and followed the co-immunoprecipitation assay by using anti-HA beads. In F, the plasmids expressing HA-tagged VEEV-TC83 capsid and TRIM32-3xF were co-transfected into 293T cells. The cells were collected at 24hours post-transfection, and followed the co-immunoprecipitation assay by using anti-HA beads. (TIF) [file ppat.1012312.s003.tif]

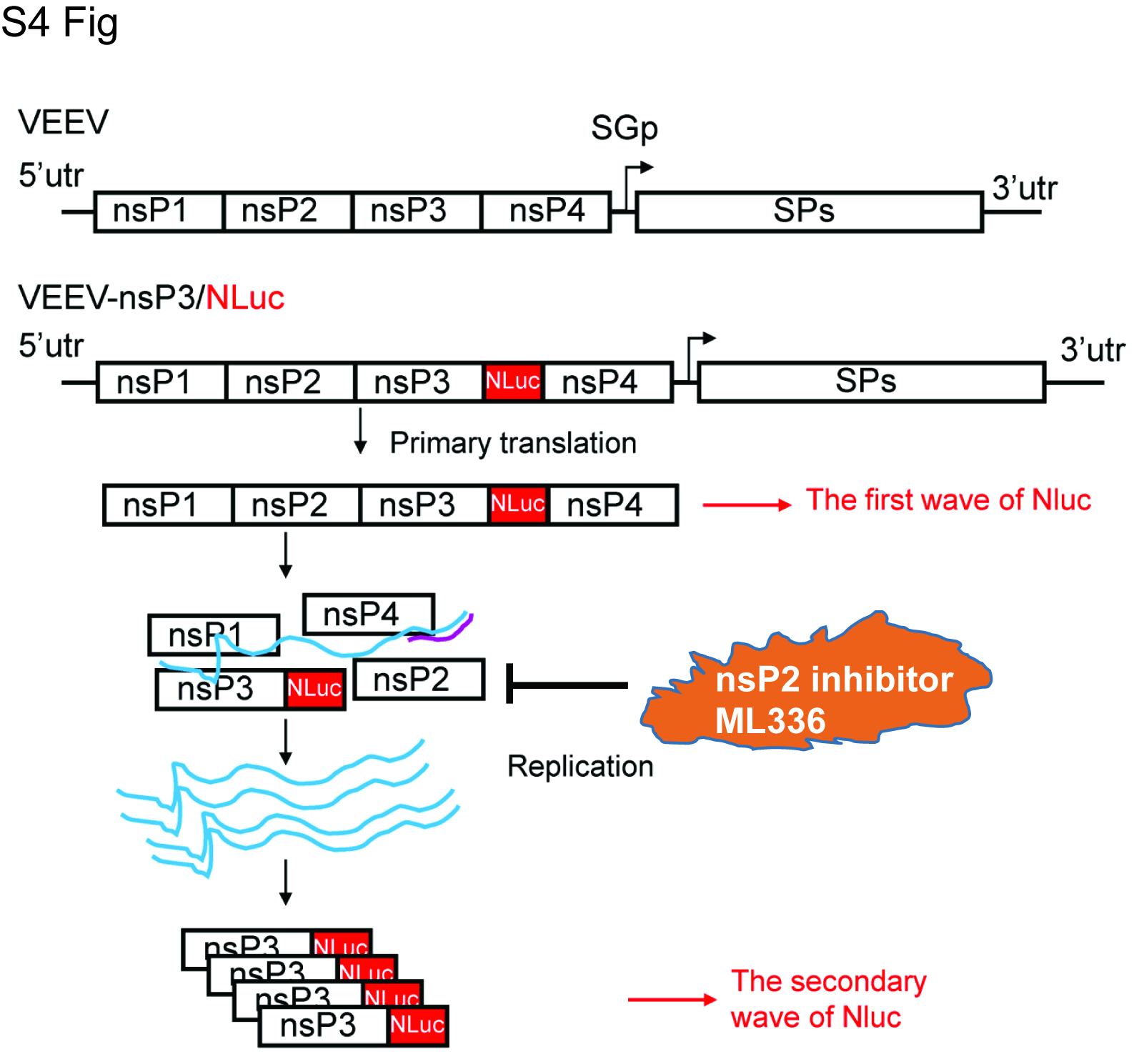

Supplement: S4 Fig — SGp: subgenomic promoter, NLuc, Nanoluciferase. (TIF) [file ppat.1012312.s004.tif]
